# Digital Health Interventions for Cardiac Rehabilitation: Systematic Literature Review

**DOI:** 10.2196/18773

**Published:** 2021-02-08

**Authors:** Shannon Wongvibulsin, Evagelia E Habeos, Pauline P Huynh, Helen Xun, Rongzi Shan, Kori A Porosnicu Rodriguez, Jane Wang, Yousuf K Gandapur, Ngozi Osuji, Lochan M Shah, Erin M Spaulding, George Hung, Kellen Knowles, William E Yang, Francoise A Marvel, Eleanor Levin, David J Maron, Neil F Gordon, Seth S Martin

**Affiliations:** 1 Johns Hopkins University School of Medicine Baltimore, MD United States; 2 University of Patras School of Medicine Patras Greece; 3 Ciccarone Center for the Prevention of Cardiovascular Disease, Division of Cardiology Department of Medicine Johns Hopkins University School of Medicine Baltimore, MD United States; 4 UCLA David Geffen School of Medicine Los Angeles, CA United States; 5 Medstar Franklin Square Hospital Baltimore, MD United States; 6 Johns Hopkins School of Nursing Baltimore, MD United States; 7 Department of Medicine Johns Hopkins Bayview Medical Center Baltimore, MD United States; 8 Department of Medicine, Division of Cardiology Stanford University School of Medicine Stanford, CA United States; 9 Stanford Prevention Research Center Stanford University School of Medicine Stanford, CA United States; 10 INTERVENT International Savannah, GA United States; 11 Centre for Exercise Science and Sports Medicine School of Therapeutic Sciences, Faculty of Health Sciences University of the Witwatersrand Johannesburg South Africa

**Keywords:** cardiac rehabilitation, telemedicine, digital technologies, mHealth, mobile phone

## Abstract

**Background:**

Cardiovascular disease (CVD) is the leading cause of death worldwide. Despite strong evidence supporting the benefits of cardiac rehabilitation (CR), over 80% of eligible patients do not participate in CR. Digital health technologies (ie, the delivery of care using the internet, wearable devices, and mobile apps) have the potential to address the challenges associated with traditional facility-based CR programs, but little is known about the comprehensiveness of these interventions to serve as digital approaches to CR. Overall, there is a lack of a systematic evaluation of the current literature on digital interventions for CR.

**Objective:**

The objective of this systematic literature review is to provide an in-depth analysis of the potential of digital health technologies to address the challenges associated with traditional CR. Through this review, we aim to summarize the current literature on digital interventions for CR, identify the key components of CR that have been successfully addressed through digital interventions, and describe the gaps in research that need to be addressed for sustainable and scalable digital CR interventions.

**Methods:**

Our strategy for identifying the primary literature pertaining to CR with digital solutions (defined as technology employed to deliver remote care beyond the use of the telephone) included a consultation with an expert in the field of digital CR and searches of the PubMed (MEDLINE), Embase, CINAHL, and Cochrane databases for original studies published from January 1990 to October 2018.

**Results:**

Our search returned 31 eligible studies, of which 22 were randomized controlled trials. The reviewed CR interventions primarily targeted physical activity counseling (31/31, 100%), baseline assessment (30/31, 97%), and exercise training (27/31, 87%). The most commonly used modalities were smartphones or mobile devices (20/31, 65%), web-based portals (18/31, 58%), and email-SMS (11/31, 35%). Approximately one-third of the studies addressed the CR core components of nutrition counseling, psychological management, and weight management. In contrast, less than a third of the studies addressed other CR core components, including the management of lipids, diabetes, smoking cessation, and blood pressure.

**Conclusions:**

Digital technologies have the potential to increase access and participation in CR by mitigating the challenges associated with traditional, facility-based CR. However, previously evaluated interventions primarily focused on physical activity counseling and exercise training. Thus, further research is required with more comprehensive CR interventions and long-term follow-up to understand the clinical impact of digital interventions.

## Introduction

### Cardiac Rehabilitation

Cardiovascular disease (CVD) is the leading cause of death worldwide, with approximately 80% of CVD resulting from modifiable risk factors such as physical inactivity, poor dietary habits, elevated low-density lipoprotein-cholesterol and plasma glucose levels, and smoking [1]. Following a cardiac event, cardiac rehabilitation (CR) is an effective modality that enhances recovery, reduces cardiovascular mortality and risk for hospital admissions, and improves the health-related quality of life (QoL) [2]. CR is a multi-faceted, medically supervised program that addresses established core components of guideline-directed therapy, including baseline patient assessments, nutritional counseling, risk factor modification (including management of lipids, blood pressure, weight, diabetes mellitus, and smoking), psychosocial interventions, and physical activity counseling and exercise training [3]. Although there is strong evidence supporting the benefits of CR, less than 20% of patients who are eligible participate in CR [4]. Challenges related to the low utilization of CR include the lack of referral or facilitation of enrollment, limited health insurance coverage, time and costs associated with participation and travel, and lack of access to a CR facility because of scheduling, transportation, or distance [5].

### Digital Technology for CR

The technology for CR is advancing rapidly and has the potential to address the challenges of traditional facility-based CR programs by delivering care to patients in the convenience of their own homes with real-time, personalized support. As noted in the literature, the terminology describing this technology has not been standardized and includes *telemedicine*, *telehealth*, and *eHealth* [6,7]. In this review, we use the term *digital health interventions* to encompass technology that enables the delivery of care through means such as the use of the internet, wearable devices, and mobile apps [8,9]. Although there have been encouraging results from the use of digital health interventions for CR (eg, remote electrocardiographic monitoring and mobile or web portal tools), these developments have largely remained in the research settings and have not yet translated into widespread use in clinical practice [3]. Currently, there are gaps in understanding the comprehensiveness of digital CR programs and how successful they are in addressing the core components of CR. To help guide the development of digital CR interventions that have the potential to translate into clinical use, we have focused on the evaluation of technology used in digital interventions for CR and the comprehensiveness of these programs using the framework outlined in the scientific statement from the American Heart Association (AHA) and the American Association of Cardiovascular and Pulmonary Rehabilitation (AACVPR) for the core components of CR [3]. The specifics regarding the accreditation of CR programs are beyond the scope of this review. With the increasing need for technological advancements to revolutionize the delivery of CR care, this systematic literature review: (1) summarizes the current literature on digital interventions for CR, (2) identifies the key components of CR that have been successfully addressed through digital interventions, and (3) describes the gaps in research that need to be addressed for the sustainable implementation of digital CR interventions in clinical practice.

## Methods

### Overview

**Figure 1:**
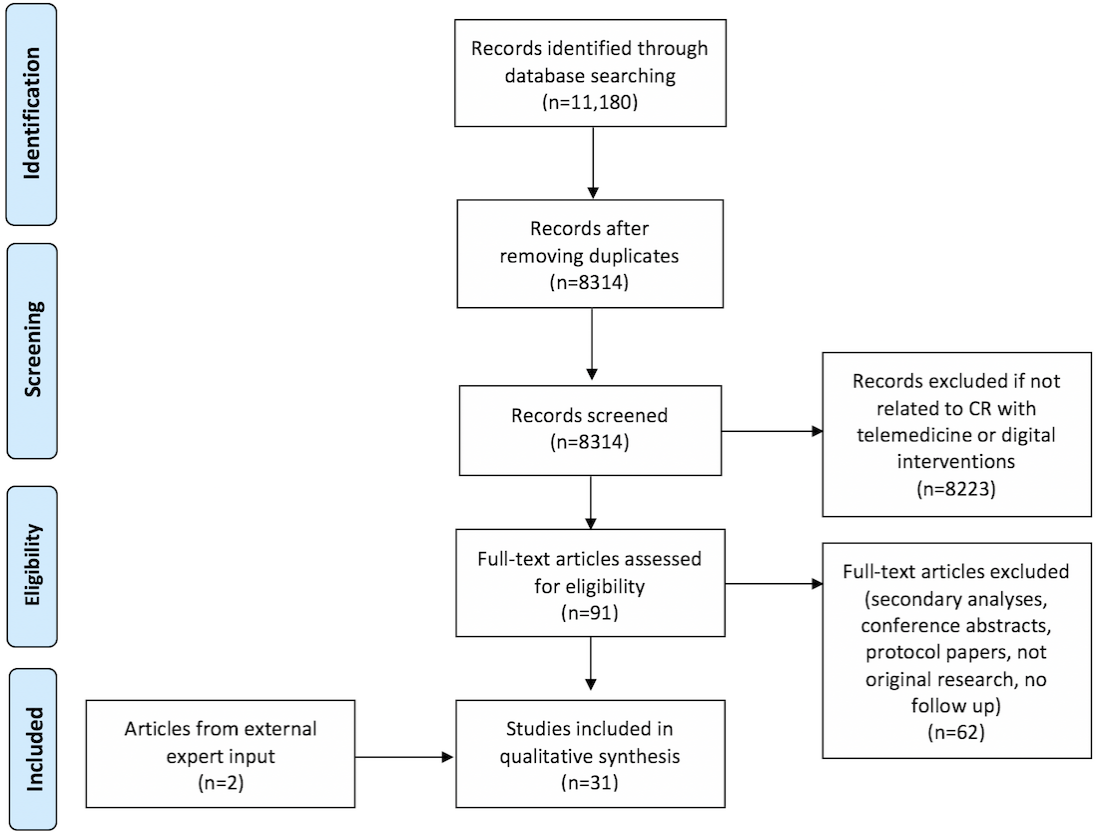
Flowchart for study identification, screening, eligibility, and inclusion. CR: cardiac rehabilitation.

We designed a systematic, thematic review to answer key questions regarding the study designs to evaluate CR interventions, technology used, study size, and comprehensiveness of the investigated interventions. A full list of questions is provided in Textbox 1. Our search terms are detailed in Multimedia Appendix 1. We searched the PubMed (MEDLINE), Embase, CINAHL, and Cochrane databases for studies on digital CR published in English between January 1, 1990, and October 18, 2018. For this review, *digital* is defined as technology employed to deliver remote care beyond the use of telephone (eg, the delivery of care using the internet, wearable devices, and mobile apps). Telephonic-only studies, which have been addressed in the 2019 scientific statement on home-based CR from the AACVPR, AHA, and American College of Cardiology (ACC) [10], are not within the scope of this review. To determine eligibility for inclusion in this study, titles and abstracts were screened for relevance before a full-text review. The inclusion criteria for this review were as follows: (1) original research study using digital or telemedicine approaches for CR and (2) reported results for feasibility, usability, or clinical outcomes. Studies were excluded if they (1) were not full-length publications (ie, abstracts), (2) were methods papers, (3) described only the technology without any inclusion of study participants, or (4) did not include any follow-up time to study outcomes (ie, cross-sectional studies). Given the evolving terminology surrounding digital health technology, we sought external expert inputs to include articles that were not found through our primary search strategy. Papers were included if they reported original research in digital CR. Full details are presented in Figure 1.

**Key questions to evaluate digital cardiac rehabilitation programs.**

1. Which study designs were employed to evaluate the digital cardiac rehabilitation (CR) interventions?
2. Which technologies were used?
3. In which countries were these studies performed?
4. What were the study sample sizes?
5. What were the durations of the interventions and follow-up times?
6. What were the findings of these digital CR intervention studies?
7. How comprehensive were the digital CR interventions?

### Evaluation of CR Components and Study Quality

For each study, we recorded the components of CR that were delivered as described in the AHA and AACVPR consensus statement on the core components of CR and categorized the digital intervention listed in each study as either standalone or adjunctive to conventional CR. Studies were designated as standalone interventions when the program was delivered remotely with the exception of initial in-person session(s) for onboarding or baseline or outcome assessments, as long as the rest of the intervention was remote. The quality of the articles was independently assessed by 2 evaluators using the National Institutes of Health: National Heart, Lung, and Blood Institute (NHI: NHLBI) Study Quality Assessment Tools, which include the evaluation of 14 criteria for an overall quality assessment of good, fair, or poor (Multimedia Appendices 2 and 3) [11]. Discrepancies in ratings were resolved by discussions between the evaluators to reach a consensus on the ratings. We followed the guidelines outlined in the Preferred Reporting Items for Systematic Reviews and Meta-Analyses [12].

## Results

### Study Characteristics

In total, 31 studies met the eligibility criteria and were included in this review (Table 1). The study characteristics are summarized in Table 2 and Multimedia Appendix 4. The median sample size was 98 (IQR 52.5-146), the median intervention duration was 3 months (IQR 1.6-4.4 months), and the median follow-up time was 6 months (IQR 3-6 months). The majority of these studies were conducted in Europe (12/31, 39%) and North America (8/31, 26%). A total of 22 studies (22/31, 71%) were randomized controlled trials. Of the 31 studies, 15 (15/31, 48%) were standalone digital CR interventions. The study quality was variable, with 23 studies (23/31, 74%) having a good quality, 7 (7/31, 23%) having a fair quality, and 1 (1/31, 3%) having a poor quality, according to the criteria established by the NIH: NHLBI Quality Assessment Tools (Multimedia Appendix 5).

**Figure 2:**
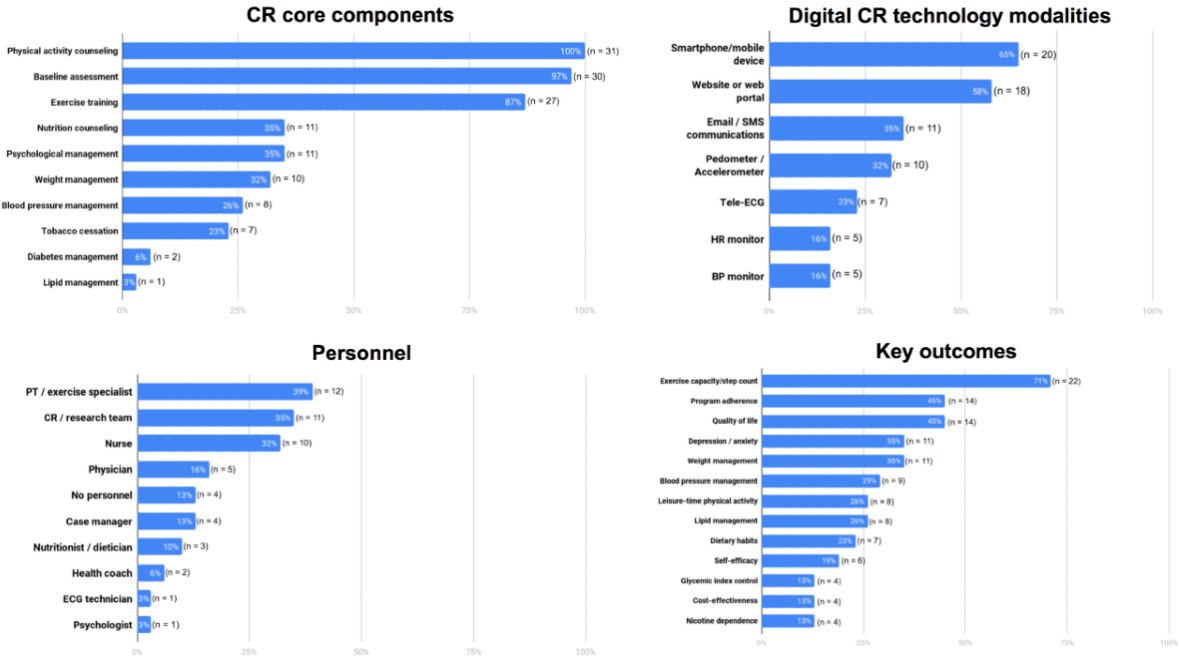
Percentage of studies with (1) the cardiac rehabilitation (CR) core components being addressed through digital interventions (apart from baseline assessment, which was conducted in person), (2) the technology modalities used in digital CR interventions, (3) the types of personnel employed in each CR program, and (4) the key outcomes evaluated. Cumulative percentages in some instances exceed 100% because some studies looked at multiple traits (n=31). BP: blood pressure; CR: cardiac rehabilitation; ECG: electrocardiogram; HR: heart rate; PT: physical therapist.

As shown in Figure 2, the most commonly targeted CR core components were physical activity counseling (31/31, 100%), baseline assessment (30/31, 97%), and exercise training (27/31, 87%). Only about one-third of the studies addressed each of the other CR core components of nutrition counseling (11/31, 35%), psychological management (11/31, 35%), and weight management (10/31, 32%). In contrast, less than a third of the studies addressed other CR core components, with only a single study including lipid management (1/31, 3%), 2 studies including diabetes management (2/31, 6%), 7 studies including tobacco cessation (7/31, 23%), and 8 studies including blood pressure management (8/31, 26%). Smartphones/mobile devices and wearables were employed in 65% (20/31) of the studies, websites or web portals in 58% (18/31), and email-SMS communications in 35% (11/31) of the studies. The interventions were most commonly guided by physical therapists or exercise specialists (12/31, 39%), followed by CR/research team staff (11/31, 35%), and nurses (10/31, 32%). Four studies (4/31, 13%) described their interventions as fully automated or did not indicate requirement of any specific personnel [13-16]. The most commonly evaluated outcome was exercise capacity or step count (22/31, 71%). Other frequently assessed outcomes included program adherence (14/31, 45%) and QoL (14/31, 45%).

**Table 1:** Characteristics of the included studies.

Reference	Quality	Country	Design and population	Intervention	CR^a^ components delivered^b^	Personnel and delivery setting^c^	Key outcomes
Ades, 2000 [17]	Fair	United States	Nonrandomized trial of patients with ACS^d^ within past 3 months	3-month home-based, transtelephonically monitored CR, compared with conventional CR	BA^e^ PAC^f^ ET^g^	Nurse coordinator IPS^h^ SAI^i^	Exercise capacity QoL^j^
Jenny, 2001 [13]	Fair	China	RCT^k^ among cardiac patients enrolled in CR	30-min interactive computer-based health education program, compared to conventional health tutorial sessions	BA PAC	Personnel required not specified Program delivered through desktop or laptop computer	Exercise self-efficacy Exercise knowledge
Gordon, 2002 [18]	Good	United States	RCT among CAD^l^ patients	12-week physician-supervised, nurse-case-managed cardiovascular risk reduction program and a community-based cardiovascular risk reduction program (including counseling via the telephone and internet) to patients with low-to-moderate-risk CAD as compared to contemporary phase II CR program	BA NC^m^ WM^n^ LM^o^ TC^p^ PAC ET PM^q^	Physician-supervised program: physician, nurse-case manager IPS Community-based program: exercise physiologists, nonphysician Health care professionals Physicians IPS	Maximal oxygen uptake BP Weight Lipid profile Medication use
Southard, 2003 [19]	Good	United States	RCT among CVD^r^ patients	6-month internet-based program containing risk factor management support, education, and monitoring services to patients with CVD, as compared to usual care	BA NC BPM PAC ET	Case manager, dietician IPS SAI	Satisfaction Participation Cost-effectiveness Weight BP Lipid profile Depression Exercise capacity Dietary habits
Barnason, 2009 [14]	Good	United States	RCT among CABG^s^ patients	6-week symptom management telehealth intervention comprised of questionnaires, accelerometer, activity diary compared to standard of care	BA PAC ET	None specified	Exercise capacity
Scalvini, 2009 [20]	Good	Italy	Pilot study of patients with postop CABG or valve surgery	1-month home-based CR with remotely transmitted ECGs^t^	BA PAC ET	Nurse-tutor, physiotherapist GS^u^ IPS SAI	Step count
Piotrowicz, 2010 [21]	Fair	Poland	RCT among patients with HF^v^	8-week home-based telemonitored CR, compared with conventional CR	BA WM BPM^w^ PAC ET PM	Physician, physiotherapist ECG technician, psychologist IPS SAI	Intervention adherence Exercise capacity
Reid, 2011 [22]	Good	Canada	RCT among CHD patients not participating in CR	6-month web-based tailored exercise intervention with email coaching, compared to standard of care	BA PAC ET	Exercise specialist IPS SAI	Exercise capacity
Clark, 2013 [23]	Fair	Australia	Pilot study of patients with post-MI^x^ or angioplasty	7-week web-based CR intervention with educational materials, workbooks, and discussion forums, glucose and BP^y^ monitoring, and pedometer	BA NC WM BPM DM^z^ TC PAC PM	General practitioner, nurse, allied health professional, case manager IPS SAI	Engagement
Brough, 2014 [24]	Fair	United Kingdom	Pilot study of patients with CHD^aa^ referred for CR	8-week web-based CR comprised of web-based coaching and exercise e-diary	BA NC WM TC PAC ET PM	CR specialist IPS SAI	Exercise capacity Nutrition Psychosocial well-being
Devi, 2014 [25]	Good	England	RCT among patients with CHD	6-week web-based CR comprised of exercise diary and web-based coaching, compared to standard of care	BA NC WM TC PAC ET PM	Researcher IPS SAI	Step count Exercise capacity Weight BP Body fat percentage QoL measures Self-efficacy Anxiety or depression Dietary habits
Forman, 2014 [26]	Good	United States	Pilot study of patients enrolled in CR	30-day task-based smartphone CR intervention comprised of medication and walking reminders, surveys, and educational tools, with web-based monitoring	PAC ET PM	Nurse manager, exercise physiologist, nutritionist	Engagement
Kraal, 2014 [27]	Good	Netherlands	RCT among low- to moderate-risk CR patients	12-week home-based CR with telemonitored coaching interventions compared to standard of care	BA PAC ET	Physical therapist IPS	Exercise capacity QoL
Piotrowicz, 2014 [28]	Poor	Poland	Nonrandomized trial of CVD patients referred for outpatient phase II CR	4-week home-based CR with remote ECG monitoring with mobile phone transmission	BA PAC ET	Nurse IPS SAI	Intervention adherence Satisfaction Exercise capacity
Varnfield, 2014 [29]	Fair	Australia	RCT among post-MI patients referred to CR	6-week home-based CR using smartphone interventions (educational materials, exercise monitoring, weekly coaching), compared with conventional CR	BA NC WM BPM TC PAC ET PM	Mentor (health coach) IPS SAI	Intervention adherence QoL Exercise capacity Weight
Whittaker, 2014 [30]	Fair	Australia	RCT among patients at post-MI	6-week home telehealth-based CR comprising mobile phone, Wellness Diary and web portal with tele-coaching, as compared to hospital-based CR	BA PAC ET	Health coach IPS SAI	Health outcomes Efficacy Participation Cost-effectiveness
Pfaeffli Dale, 2015 [16]	Good	New Zealand	Qualitative survey of patients with CHD	24-week mobile health program comprising text messaging and web-based coaching plus center-based CR, compared to center-based CR alone	BA NC TC PAC PM	Fully automated digital intervention IPS	Lifestyle modification QoL Intervention adherence
Frederix, 2015 [31]	Good	Belgium	RCT among CAD patients who completed phase II CR	18-week telemonitored exercise program, compared with standard of care	BA PAC ET	Rehabilitation center staff IPS	Exercise capacity Weight Lipid profile Glycemic control Rehospitalizations
Lear, 2015 [32]	Good	Canada	RCT among patients with ACS or postrevascularization	4-month web-based CR comprising education, coaching, and physiologic data monitoring, compared with standard of care	BA NC WM BPM DM PAC ET	Program nurse, case manager, exercise specialist, dietician GS IPS SAI	Exercise capacity Lipid profile Dietary outcomes
Maddison, 2015 [15]	Good	New Zealand	RCT among patients with IHD^ab^	24-week smartphone-based intervention (website, educational videos, text messaging) plus standard of care, compared with standard of care alone	BA NC PAC ET PM	Not specified	Exercise capacity QoL Cost-effectiveness
Smolis-Bak, 2015 [33]	Good	Poland	Prospective randomized study among patients with HF and implanted CRT-D^ac^	8-week telemonitored home-based CR, compared to no training program after discharge	BA PAC ET	CR center staff, physiotherapist, doctor, nurse IPS	Exercise capacity Echo evaluation QoL
Frederix, 2016 [34]	Good	Belgium	Cost-effectiveness analysis of patients with CR	24-week web-based telerehabilitation program (web-based coaching, accelerometer) plus CR, compared to CR alone	BA NC TC PAC ET	Cardiac nurse, rehabilitation nurse IPS	Cost-effectiveness Rehospitalizations
Skobel, 2016 [35]	Good	Germany Spain United Kingdom	RCT among patients with CAD referred for CR	6-month smartphone-based exercise intervention (remote monitoring, physiologic data capture, and coaching), as compared to conventional CR	BA PAC ET	Sports physicians, exercise scientists IPS SAI	Exercise capacity
Thorup, 2016 [36]	Good	Denmark	RCT among hospitalized patients with ACS, HF, or coronary bypass surgery	3-month telerehabilitation trial with pedometer, compared among 3 rehabilitation settings	BA WM BPM PAC	Personal nurse GS IPS	Step count
da Silva Vieira, 2017 [37]	Good	Portugal	RCT among patients who completed CR	6-month virtual reality CR intervention (Kinect) or booklet CR intervention, compared with standard of care	BA PAC ET	Researchers IPS	Body composition Eating patterns Lipid profile
Hwang, 2017 [38]	Good	Australia	RCT among stable patients with chronic HF	12-week home-based CR with web-based video conferencing, compared with facility-based CR	BA NC PAC ET PM	Research staff, physiotherapists GS IPS SAI	Exercise capacity QoL
Fang, 2018 [39]	Good	China	RCT among patients at post-PCI^ad^	6-week home-based CR with remote physiological monitoring and education, as compared with conventional CR	BA PAC ET	Medical team IPS	Exercise capacity BP QoL Nicotine dependence
Harzand, 2018 [40]	Good	United States	Pilot study of veterans with CHD and eligible for CR	12-week home-based CR with smartphone app utilizing exercise reminders, educational materials, vitals monitoring, and remote coaching	BA WM BPM PAC ET	CR coach (cardiology physician assistant) SAI	Feasibility BP Acceptability Exercise capacity
Maddison, 2018 [41]	Good	New Zealand	RCT among patients with CHD	12-week remotely monitored telerehabilitation with coaching, compared with conventional CR	BA PAC ET	CR exercise specialist IPS	Exercise capacity QoL Intervention adherence
Peng, 2018 [42]	Good	China	RCT among patients with HF	8-week home-based CR with remote coaching using physiologic data capture, web-based portal, and smartphone, compared with standard of care	BA WM BPM PAC ET PM	Multidisciplinary CR team IPS	Exercise capacity QoL Echo evaluation
Rawstorn, 2018 [43]	Good	New Zealand	RCT among patients with CHD eligible for CR	12-week remotely monitored telerehabilitation with coaching, as compared with conventional CR	BA PAC ET	Exercise specialist	Usability Satisfaction

^a^CR: cardiac rehabilitation.

^b^CR components were delivered through digital interventions except for baseline assessment that were conducted in person.

^c^Delivery setting: group sessions (GS), in-person session (IPS), standalone intervention (SAI).

^d^ACS: acute coronary syndrome.

^e^BA: baseline assessment.

^f^PAC: physical activity counseling.

^g^ET: exercise training.

^h^IPS: in-person session.

^i^SAI: standalone intervention.

^j^QoL: quality of life.

^k^RCT: randomized controlled trial.

^l^CAD: coronary artery disease.

^m^NC: nutrition counseling.

^n^WM: weight management.

^o^LM: lipid management.

^p^TC: tobacco cessation.

^q^PM: psychological management.

^r^CVD: cardiovascular disease.

^s^CABG: coronary artery bypass grafting.

^t^ECG: electrocardiogram.

^u^GS: group session.

^v^HF: heart failure.

^w^BPM: blood pressure management.

^x^MI: myocardial infarction.

^y^BP: blood pressure.

^z^DM: diabetes management.

^aa^CHD: coronary heart disease.

^ab^IHD: ischemic heart disease.

^ac^CRT-D: cardiac resynchronization therapy with defibrillator function.

^ad^PCI: percutaneous coronary intervention.

**Table 2:** Summary of the studies included in the analysis (n=31).

Characteristics		Value
**Type of study, n (%)**		
	RCT^a^	22 (71)
	Pilot study	5 (16)
	Nonrandomized trial	2 (6)
	Cost-effectiveness analysis from RCT	1 (3)
	Qualitative study	1 (3)
**Location of study, by continent, n (%)**		
	Europe	12 (39)
	North America	8 (26)
	Australia (including New Zealand)	8 (26)
	Asia	3 (10)
**Publication year, n (%)**		
	1990-2000	1 (3)
	2001-2010	6 (19)
	2011-2018	24 (77)
Median sample size (IQR)		98 (52.5-146)
Median follow-up time (months; IQR)		6 (3-6)
Median intervention duration (months; IQR)		3 (1.6-4.4)

^a^RCT: randomized controlled trial.

### Key Outcomes

The key findings from the 31 studies are summarized in Textbox 2. Overall, program adherence was greater in patients using digital interventions than in those participating in conventional CR. Moreover, digital CR interventions were comparable to conventional CR (control) groups across multiple short-term outcomes (eg, functional capacity, physical activity, self-efficacy, program adherence, weight management, dietary habits, and QoL). However, digital CR interventions had mixed efficacy with regard to blood pressure control and mood. Of the 9 studies reporting blood pressure as an outcome of digital interventions versus conventional CR, 3 studies showed noninferiority [18,29,41], 4 found no significant impact on blood pressure [19,28,31,39], and 2 reported a better control of blood pressure in the control group as compared to the digital intervention group [25,35]. Similarly, although the majority of studies assessing mood as an outcome reported that digital interventions were noninferior to conventional CR in improving mood [22,25,29,30,33], 4 studies reported no significant improvement in anxiety or depression [19,24,25,42].

**Summary of findings by thematic outcomes.**

Blood glucose control

- Only 1 of the 4 studies reported an improvement in glycemic control in the intervention group [31]. In total, 2 studies found no significant impact of the digital cardiac rehabilitation (CR) intervention on glycemic control [29,35]. One study found no significant difference in glycemic control between the digital intervention and usual care groups [41].

Blood pressure management

- In total, 3 of the 9 studies described CR interventions that significantly improved blood pressure management and were noninferior to the control groups [18,29,41]. A total of 4 studies did not find the digital CR interventions to significantly influence blood pressure management [19,28,31,39]. In all, 2 studies found that the control group had better blood pressure management as compared to the intervention group [25,35].

Depression or anxiety

- In total, 5 studies reported a positive effect on mood in the intervention group [22,25,29,30,33]. A total of 4 studies found that digital interventions had no significant impact on mood [19,24,25,42]. In total, 2 studies found no significant change in the psychological status between digital intervention and usual care groups [35,39]. One study found that both the intervention and usual care groups experienced an improvement in mood as compared with baseline, and there was no significant difference between the intervention and control groups [39]. One study found a negative effect on depression in the intervention group [16]. Of note, Devi et al [25] found that mood improved in the short term but was not significantly different from baseline at 6 months.

Dietary habits

- In total, 5 of the 7 studies found improvement in dietary habits [24,29,30,32,37], whereas 2 studies found that the intervention had no significant impact on the participants’ dietary habits [19,25].

Exercise capacity

- In total, 22 studies looked at functional capacity as an outcome, and all of them reported that the intervention group was not inferior to the control group [15,17-22,24,25,27-33,35,38-42]. Of note, 4 studies found no significant difference in the functional capacity between the digital intervention and usual care groups [19,31,33,41]. Furthermore, Smolis-Bak et al [33] found functional capacity at the 12-month follow-up to be comparable to that at the baseline visit.

Lipid management

- In total, 2 of the 8 studies reported an increase in high-density lipoprotein in the intervention group [31,37], 2 of the 8 reported decreased low-density lipoprotein and total cholesterol [18,32], and 2 of the 8 studies reported a decrease in triglycerides [29,30].

Nicotine dependence

- Only 1 [39] of the 4 studies showed an improvement in smoking habits as measured by the Fagerstrom Test for Nicotine Dependence score [18,19,24,39].

Physical activity

- All 8 studies examining physical activity found that there was an improvement in the intervention group, comparable to or even greater than the increase in the physical activity in the control group [14,15,22,25,31,32,36,41].

Program adherence

- All 14 studies evaluating program adherence to CR reported that adherence to digital interventions was not inferior to traditional interventions [19-21,26-30,35,37,38,40,41,43]. Of note, 9 of the 14 studies found adherence to be greater in the digital intervention group [20,21,26,28-30,35,38,40].

Quality of life (QoL)

- In total, 10 studies reported an improvement in QoL in the intervention group [15,17,22,24,25,27,29,33,39,42]. A total of 6 studies found no significant difference in QoL measures between the digital intervention and usual care groups [17,21,27,35,38,41].

Self-efficacy

- Of the 6 studies evaluating self-efficacy, 5 showed improvement in self-efficacy following the digital interventions [13,15,25,32,41]. Pfaeffli Dale et al [16] found that the digital CR intervention had no significant impact on the participants’ overall self-efficacy.

Weight management

- Digital CR interventions effectively addressed weight management in 8 of the 11 studies [18,19,25,28-30,37,41]. A total of 2 studies found no difference in weight or body mass index before and after the digital CR intervention [24,35]. In one study [17], the home group had increased weight, whereas the on-site control group had a slightly decreased weight.

We found a paucity of studies specifying intervention components that targeted lipid management, glycemic index control, and smoking cessation. These components were often reported as secondary outcome measures, if at all. Furthermore, the research on the long-term efficacy of digital CR interventions was sparse with heterogeneous findings. Although Devi et al [25] found improvement in outcomes such as QoL, self-efficacy, and physical activity in the short term, no significant intervention effect was present on these outcomes when assessed at the 6-month follow-up. However, they noted that the intervention group demonstrated trends of improved levels of physical activity, whereas the control group did not. Reid et al [22] also reported long-term improvements in self-reported QoL and physical activity as long as 12 months from the start of the digital CR program, which was delivered over a 6-month period.

The examination of the studies by follow-up time revealed that the majority of the key outcome findings were mixed. However, all the studies reporting outcomes regarding adherence [19-21,26-30,35,37,38,40,41,43], QoL [15,17,21,22,24,25,27,29,33,35,38,39,41,42], and exercise capacity [15,17-22,24,25,27-33,35,38-42] found positive results or outcomes that were noninferior to the control group. Only studies with a follow-up period longer than 3 months reported outcomes for physical activity [14,15,22,25,31,32,36,41] and blood glucose control [29,31,35,41]. Regarding physical activity, the results were positive [14,15,22,25,31,32,36,41], whereas the results regarding blood glucose management were mixed, with positive effects [31], nonsignificant effects [29,35], or comparable results between the intervention and the control groups [41]. Similarly, the outcomes for blood pressure, depression or anxiety, and weight management were mixed (with positive effects, nonsignificant effects, or comparable results between the intervention and control groups) in both studies with shorter (3 months or less) [18,24,28,39] and longer (more than 3 months) [19,22,25,29-31,33,35,37,41,42] follow-up times. The only exceptions were as follows: one study reported a negative impact on mood at 6 months [16], 2 studies reported that the control group had better blood pressure management than the intervention group [25,35], and one study found an increase in body weight in the intervention group at 3 months [17]. For studies reporting outcomes regarding dietary habits, lipid management, and self-efficacy, studies with longer than a 3-month follow-up period reported positive or nonsignificant effects [15,16,19,25,29-32,35,37,41] or outcomes that were comparable between the intervention and the control groups [35,41], whereas studies with a follow-up period of 3 months or less found positive results [13,18,24]. The outcomes for nicotine dependence were mixed (positive or nonsignificant effects) among the studies with a short follow-up period [18,24,39], whereas one study reported no impact on smoking at 6 months [19].

The examination of more comprehensive, standalone interventions revealed that only 6 studies included in this review were standalone interventions delivering 5 or more CR components (other than a baseline assessment) [21,23-25,29,32]. The results of these studies for the majority of the key outcomes related to the CR components delivered through the interventions were heterogeneous. Of these 6 more comprehensive standalone interventions, only Brough et al [24] reported the outcomes regarding nicotine dependence, finding no impact on smoking. Devi et al [25] and Brough et al [24] found that their digital interventions had no significant impact on mood, whereas Varnfield et al [29] reported a positive effect on mood and anxiety levels in the intervention groups. Regarding dietary habits, Varnfield et al [29], Lear et al [32], and Brough et al [24] found improvement in the dietary habits of the intervention group, whereas Devi et al [25] found no significant impact. Three of these studies [24,25,29] reported an improvement in QoL in the intervention group, but Piotrowicz et al [21] found no significant difference in QoL measures between the intervention and control groups. Five of these studies reported a positive effect of digital intervention on exercise capacity [21,24,25,29,32]. In terms of weight management, Devi et al [25] and Varnfield et al [29] found a positive impact on weight in the intervention group, whereas Brough et al [24] found no impact on weight in the intervention group. Overall, there was a wide variety in the interventions delivered and outcomes reported.

## Discussion

### The Potential of Digital CR

This study highlights digital technology as a potential means of enhancing care and broadening access to CR through tailored interactive interventions. Our work differs from previous systematic reviews as our emphasis is on digital CR interventions with a focus on providing a systematic evaluation of the current literature to better understand the characteristics of these interventions. This study builds upon a growing body of literature supporting the use of internet-based features such as web portals and digital devices (eg, wearables) to remotely deliver CR components.

We found that digital CR was feasible and as effective as traditional CR in improving outcomes, whether as an adjunct or as an alternative to traditional CR [16,21,26,29,39,41,42]. Our findings support the conclusions of a previous study demonstrating a similar effectiveness of home- and center-based CR in improving clinical and health-related QoL outcomes in patients with myocardial infarction, myocardial revascularization, and heart failure [44]. In addition, Huang et al found that telehealth CR interventions were noninferior to center-based CR, both in the short term (12 weeks-1 year) and long term (up to 6 years), when comparing participants’ exercise capacity, all-cause mortality, and modifiable risk factors, including blood pressure, blood lipids, smoking, and weight [45]. Moreover, the AACVPR, AHA, and ACC recently released a consensus statement highlighting evidence that home- and facility-based CR can achieve similar improvements in 3-12–month clinical outcomes [10]. These developments highlight digital technology as a potential means of enhancing care and broadening access to CR through tailored interactive interventions [46,47]. However, our study and literature indicate that until digital CR is further developed and better understood, there will be a need for in-person CR sessions (Multimedia Appendix 6). In-person sessions may help digital CR by establishing baseline and monitoring progress, personalizing treatment plans, and bridging patient technology-usage challenges through technology education and deployment, especially for older users [48-50]. Currently, there are several ongoing clinical trials that are studying the efficacy of digital CR interventions [51-54].

### Key Recommendations for Future Research

A limitation of our study is the heterogeneity of the identified papers, thus prohibiting meta-analysis. Papers varied in CR technologies, interventions, study design, measured outcomes, and control groups. For example, when considering a traditional CR population as a control group, some studies used standard care, some used direct comparison with facility-based CR, and other feasibility studies did not have a control comparison group. Although the diversity in studies proved challenging to quantify, it reiterates the motivation of this systematic review: digital health-based CR is emerging as an alternative or adjunct to standard CR; thus, methodologies have yet to reach a consensus. Consequently, we encourage practitioners to study digital CR approaches in a collaborative environment to promote the standardization and optimization of study methods. We have summarized our key recommendations in Textbox 3.

**Key recommendations for researchers conducting digital cardiac rehabilitation studies.**

- Clearly state the specific goals of the intervention: whether it is designed to be a comprehensive standalone program or to be used adjunctively with traditional cardiac rehabilitation (CR)
- Describe the specifics of the CR components targeted through the intervention and the technology, equipment, or personnel required to deliver each of the components
- Include the specific details of the comparison group (eg, elaborate on what *usual care* consists of and include information about the specific intervention the comparison group received)

Beyond the heterogeneity of the study designs, we identified other limitations. Although we evaluated CR program components using the AHA and AACVPR’s consensus statement on the core components of CR, the CR programs outside of the United States may differ from professional society guidelines in the United States. In addition, some studies included only minimal details about the specific components of their interventions; thus, the number of CR components delivered may have been greater than that captured in this review. Although studies reported the technology used (summarized in Multimedia Appendix 7), specific details were often sparse, limiting the evaluation of specifically what worked well and what was difficult to implement. Furthermore, the studies did not address long-term outcomes, as the maximum reported follow-up was 16 months, and most studies had fairly small sample sizes. Finally, the majority of the studies reviewed included sessions that were conducted in person for at least a portion of the overall CR intervention.

We identified several potential directions for future research. Although digital interventions have been found to successfully deliver the components of CR pertaining to physical activity, there remains a paucity of comprehensive digital CR program interventions that address risk factors such as lipid management, blood glucose level control, and smoking cessation. In addition, the long-term effectiveness of digital approaches to CR requires further evaluation [8,55]. Furthermore, studies with larger sample sizes and adequate control groups for comparison are necessary to better understand the impact of these interventions. Additional studies are also required to investigate the frequency of adverse events in patients participating in these interventions compared with traditional approaches. The adverse effects reported in the studies reviewed fell into the categories of (1) cardiac related (acute coronary syndrome, stable angina, arrhythmias, pericarditis, dyspnea, syncope, etc), (2) potential cardiac etiology (pleural effusions, cerebral ischemia, etc), (3) noncardiac related (peripheral artery disease, pneumonia, accidents, etc), and (4) death. A number of studies reported no significant difference in the rate of adverse effects between the digital intervention and control groups [14,30,33,38]. There were also studies that found that the intervention group had fewer associated adverse events than the control group; however, this did not always reach statistical significance [19,22,31,32,34]. Only one study reported that the digital CR intervention group had more adverse events during the treatment period as compared with the control group that participated in a center-based CR; however, adverse events were comparable during the postintervention follow-up period [41]. In some cases, no major adverse events occurred throughout the study [17,26-28,40,42], or the adverse effects were unrelated to the study intervention [16,21,35]. No conclusions could be drawn regarding the increased or decreased risk of adverse effects in some studies [15,18,20]. Overall, our findings highlight the need for robust digital intervention study designs with more comprehensive programs and analysis of both the short- and long-term effects.

Furthermore, as briefly mentioned in the Methods section, our team consulted with an external expert to include relevant original research on digital approaches to CR that may have been inadvertently excluded from our primary search strategy. We opted for this approach given that although the field of telehealth and telemedicine has grown rapidly over the past few decades, the adoption of common terminology remains in infancy [7]. The evolution of this terminology has been demonstrated in a bibliometric analysis by Fatehi and Wootton [6], who noted the emergence of terms such as *eHealth* and *mHealth* as well as the usage trends of the terms such as *telemedicine*, *telehealth*, and *eHealth* in the literature. As a result, there may be studies that include digital components, such as the usage of the internet, which our search terms failed to capture. Our experience further highlights the importance of more standardized terminology surrounding digital health interventions and an understanding of the evolving terminology to accurately review the existing literature.

### Conclusions

Overall, we found that digital technology offers the potential to address the challenges associated with traditional, facility-based CR. If implemented on a large scale, digital CR could provide a level of impact, accessibility, affordability, cost savings, and benefits to patients not possible with conventional CR. However, so far, interventions have primarily focused on physical activity counseling and exercise training and not on the other core components of CR. In addition, our study focused on the evaluation of the technology used in digital CR and the comprehensiveness of these programs, but the intricacies of accreditation for CR programs are beyond the scope of this review. Further research is required with more comprehensive CR interventions to understand the long-term clinical impact of digital CR solutions on key cardiovascular outcomes and establish best practices for the development, delivery, and assessment of digital CR. 

## Appendix

Multimedia Appendix 1 Search Terms.

Multimedia Appendix 2 National Institutes of Health quality assessment tools: quality assessment of controlled intervention studies.

Multimedia Appendix 3 National Institutes of Health quality assessment tools: quality assessment tool for observational cohort and cross-sectional studies.

Multimedia Appendix 4 Summary of study and patient characteristics.

Multimedia Appendix 5 National Institutes of Health quality assessment for studies reviewed.

Multimedia Appendix 6 Aspects of in-person sessions.

Multimedia Appendix 7 Technology used in studies.

## Abbreviations

AACVPR: American Association of Cardiovascular and Pulmonary Rehabilitation
ACC: American College of Cardiology
AHA: American Heart Association
CR: cardiac rehabilitation
CVD: cardiovascular disease
mHealth: mobile health
NHLBI: National Heart, Lung, and Blood Institute
NIH: National Institutes of Health
NINR: National Institute of Nursing Research
QoL: quality of life
