# Supplementary material for: Digital Health Interventions for Cardiac Rehabilitation: Systematic Literature Review
Source: J Med Internet Res. 2021 Feb 8;23(2):e18773. doi: 10.2196/18773 (PMC7899799; doi:10.2196/18773)
Supplement: Multimedia Appendix 1 [file jmir_v23i2e18773_app1.docx]

**Search Terms**

**PubMed**

(“myocardial infarction”[mesh] OR “Myocardial Ischemia”[mesh] OR “coronary disease”[mesh] OR “Coronary Artery Bypass”[mesh] OR “heart bypass, left”[mesh] OR “heart bypass, right”[mesh] OR “Myocardial Revascularization”[mesh] OR “heart failure”[mesh] OR “heart  diseases”[mesh] OR coronary[tiab] OR myocard*[tiab] OR cardiac*[tiab] OR heart attack*[tiab] OR heart infarct*[tiab] OR angina[tiab] OR “heart failure”[tiab] OR heart disease*[tiab] OR CABG[tiab] OR PTCA[tiab] OR ((heart[tiab] or cardiac[tiab]) AND stent*[tiab]) OR “heart bypass”[tiab] OR postmyocard*[tiab])

AND

(rehab*[tiab] OR “rehabilitation”[mesh])

AND

(internet[tiab] Or web[tiab] OR online[tiab] OR tech*[tiab] OR mobile[tiab] OR home[tiab] OR remote[tiab] OR mhealth[tiab] OR digital[tiab] OR telehealth[tiab] OR telemedicine[tiab] OR “tele health”[tiab] OR “tele medicine”[tiab] OR telemonitor*[tiab] OR telerehab*[tiab] OR virtual[tiab] OR smartphone[tiab] Or ehealth[tiab] OR “e health”[tiab] OR “text messaging”[tiab] OR community[tiab] OR hybrid[tiab] OR “connected health”[tiab] OR wearable[tiab])

**Embase**

'heart infarction'/exp OR 'heart muscle ischemia'/exp OR 'coronary artery bypass surgery'/exp OR 'coronary artery disease'/exp  OR 'heart muscle revascularization'/exp OR 'angina pectoris'/exp OR 'heart failure'/exp OR 'heart disease'/exp OR 'extracorporeal circulation'/exp OR

(heart attack* OR coronary OR myocard* OR cardiac*OR heart infarct* OR angina OR ‘heart failure*’ OR ‘heart disease*’ OR CABG OR PTCA OR ((heart or cardiac) AND stent*) OR ‘heart bypass*’ OR postmyocard*):ti,ab)

AND

(rehab*:ti,ab OR 'rehabilitation'/exp)

AND

(internet Or web OR online OR tech* OR mobile OR home OR remote OR mhealth OR digital OR telehealth OR telemedicine OR “tele health” OR ‘tele medicine*’ OR telemonitor* OR telerehab* OR virtual OR smartphone Or ehealth OR ‘e health’ OR ‘text messaging’ OR community OR hybrid OR ‘connected health’ OR wearable):ti,ab

**Cochrane**

(“myocardial infarction”[mesh] OR “Myocardial Ischemia”[mesh] OR “coronary disease”[mesh] OR “Coronary Artery Bypass”[mesh] OR “heart bypass, left”[mesh] OR “heart bypass, right”[mesh] OR “Myocardial Revascularization”[mesh] OR “heart failure”[mesh] OR “heart  diseases”[mesh] OR coronary OR myocard* OR cardiac* OR heart attack* OR heart infarct* OR angina OR “heart failure” OR heart disease* OR CABG OR PTCA OR ((heart or cardiac) AND stent*) OR “heart bypass” OR postmyocard*)

AND

(rehab* OR “rehabilitation”[mesh])

AND

(internet Or web OR online OR tech* OR mobile OR home OR remote OR mhealth OR digital OR telehealth OR telemedicine OR “tele health” OR “tele medicine” OR telemonitor* OR telerehab* OR virtual OR smartphone Or ehealth OR “e health” OR “text messaging” OR community OR hybrid OR “connected health” OR wearable)

**CINAHL**

(MH "Myocardial Infarction") OR “myocardial infarction” OR (MH "Myocardial Ischemia") OR heart attack* OR (MH "Coronary Artery Bypass") OR coronary OR myocard* OR cardiac* OR (MH "Coronary Disease") OR (MH "Myocardial Revascularization") OR heart infarct* OR (MH "Angina Pectoris") OR angina OR (MH "Heart Failure") OR “heart failure” OR (MH "Heart Diseases") OR “heart disease*” OR CABG OR PTCA OR ((heart or cardiac) AND stent*) OR “heart bypass” OR postmyocard*)

AND

(rehab* OR (MH "Rehabilitation")

AND

(internet Or web OR online OR tech* OR mobile OR home OR remote OR mhealth OR digital OR telehealth OR telemedicine OR “tele health” OR “tele medicine” OR telemonitor* OR telerehab* OR virtual OR smartphone Or ehealth OR “e health” OR “text messaging” OR community OR hybrid OR “connected health” OR wearable)

This is a Multimedia Appendix to a full manuscript published in the J Med Internet Res. For full copyright and citation information see https://dx.doi.org/10.2196/jmir.18773
