# Supplementary material for: Digital Health Interventions for Cardiac Rehabilitation: Systematic Literature Review
Source: J Med Internet Res. 2021 Feb 8;23(2):e18773. doi: 10.2196/18773 (PMC7899799; doi:10.2196/18773)
Supplement: Multimedia Appendix 2 [file jmir_v23i2e18773_app2.docx]

**NIH Quality Assessment Tools*: Quality Assessment of Controlled Intervention Studies**

| **Quality Assessment of Controlled Intervention Studies** | |
| --- | --- |
| **1** | Was the study described as randomized, a randomized trial, a randomized clinical trial, or an RCT? |
| **2** | Was the method of randomization adequate (i.e., use of randomly generated assignment)? |
| **3** | Was the treatment allocation concealed (so that assignments could not be predicted)? |
| **4** | Were study participants and providers blinded to treatment group assignment? |
| **5** | Were the people assessing the outcomes blinded to the participants' group assignments? |
| **6** | Were the groups similar at baseline on important characteristics that could affect outcomes (e.g., demographics, risk factors, co-morbid conditions)? |
| **7** | Was the overall drop-out rate from the study at endpoint 20% or lower of the number allocated to treatment? |
| **8** | Was the differential drop-out rate (between treatment groups) at endpoint 15 percentage points or lower? |
| **9** | Was there high adherence to the intervention protocols for each treatment group? |
| **10** | Were other interventions avoided or similar in the groups (e.g., similar background treatments)? |
| **11** | Were outcomes assessed using valid and reliable measures, implemented consistently across all study participants? |
| **12** | Did the authors report that the sample size was sufficiently large to be able to detect a difference in the main outcome between groups with at least 80% power? |
| **13** | Were outcomes reported or subgroups analyzed prespecified (i.e., identified before analyses were conducted)? |
| **14** | Were all randomized participants analyzed in the group to which they were originally assigned, i.e., did they use an intention-to-treat analysis? |

*Source: https://www.nhlbi.nih.gov/health-topics/study-quality-assessment-tools

This is a Multimedia Appendix to a full manuscript published in the J Med Internet Res. For full copyright and citation information see https://dx.doi.org/10.2196/jmir.18773
