# Supplementary material for: Digital Health Interventions for Cardiac Rehabilitation: Systematic Literature Review
Source: J Med Internet Res. 2021 Feb 8;23(2):e18773. doi: 10.2196/18773 (PMC7899799; doi:10.2196/18773)
Supplement: Multimedia Appendix 4 [file jmir_v23i2e18773_app4.docx]

**Summary of Study and Patient Characteristics**

| **Author, Year** | **Age (Intervention),  mean (SD)  or median (IQR)** | **Age (Control),  mean (SD) or median  (IQR)** | **% Male  (Intervention)** | **% Male  (Control)** | **Other Patient Characteristics  Noted in Study (Intervention)** | **Other Patient Characteristics  Noted in Study (Control)** | **% Completion  (Intervention)** | **% Completion  (Control)** |
| --- | --- | --- | --- | --- | --- | --- | --- | --- |
| **Ades, 2000** | 56 +/- 9 | 58 +/- 12 | 76% | 90% | CABG: 50.6%, MI: 13.3%, PTCA: 28.9%, Transplant: 7.2% | CABG: 54%, MI: 16%, PTCA: 28%, Transplant: 2% | 91% | 92% |
| **Jenny, 2001** | 56.12 | 58.81 | 79.20% | 62.50% |  |  | 56.30% | 33.30% |
| **Gordon, 2002** | case-managed (CM):  61+/- 10, community-based (CB) program:  60 +/- 9 | 60 +/- 9 | CM: 73%, CB: 78% | 76% | Case-managed: education: college education or above: 62%, prior MI: 29%, prior CABG: 37%, prior PTCA: 42%, chronic stable angina: 10%  Community-based program: education: college education or above: 51%, prior MI: 16%, prior CABG: 40%, prior PTCA: 47%, chronic stable angina: 7% | Education: college education or above: 67%. prior MI: 16%, prior CABG: 38%, prior PTCA: 53%, chronic stable angina: 7% | 91.6% (total) | |
| **Southard, 2003** | 61.8 (10.6) | 62.8 (10.6) | 68% | 82% | Education: High school: 26.4%, Trade school or associate degree: 28.3 %, Bachelor’s degree: 22.6%, Master’s degree: 17%, PhD: 3.8%, Other: 1.9%. Myocardial infarction: 52.8%, Congestive heart failure: 13.2%, Coronary artery bypass graft: 56.6%, Percutaneous transluminal coronary angioplasty: 43.4% | Education: High school: 25.5%, Trade school or associate degree: 15.7%, Bachelor’s degree: 29.4%, Master’s degree: 19.6%, PhD: 2%, Other: 7.8%. Myocardial infarction: 62.7%, Congestive heart failure: 5.9%, Coronary artery bypass graft: 62.7%, Percutaneous transluminal coronary angioplasty: 33.3% | 92.50% | 100% |
| **Barnason, 2009** | 71.21+/- 4.91 (total) | | 82.76% (total) | | NYHA I:48.48%, NYHA II:41.13%, NYHA III:9.96%,  NYHA IV:0.43% | | 99.10% | 99.20% |
| **Scalvini, 2009** | 64 | no  control  group | 87% | no  control  group |  | no control group | 100% | no control group |
| **Piotrowicz, 2010** | 56.4 +/- 10.9 | 60.5 +/- 8.8 | 85% | 95% | NYHA II: 49.3%, NYHA III: 50.7% | NYHA II: 55.4%, NYHA III: 44.6% | 97.40% | 78.70% |
| **Reid, 2011** | 56.7 +/- 9.0 | 56.0 +/- 9.0 | 82.60% | 86.10% | Education: 14.2 years +/- 3.2, first cardiac event: 68.7%, previous MI: 15.7%, previous PCI: 25.2%, previous CABG: 8.7% | Education: 14.4 years +/- 3.1, first cardiac event: 60.2 %, previous MI: 22.2 %, previous PCI: 29.6%, previous CABG: 9.3% | 68.70% | 68.50% |
| **Clark, 2013** | 62.4 | no  control  group | 81.50% | no  control  group |  | no control group | 45.80% | no control group |
| **Brough, 2014** | 60.5 (11.1) | no  control  group | 90.20% | no  control  group | PCI: 53.7%, CABG:24.4%, Other: 4.9% Valve Surgery: 14.6%, Medical management:7.3% | no control group | 80.50% | no control group |
| **Devi, 2014** | 66.27 (8.35) | 66.20 (10.06) | 71% | 78% | Angina treatment- Medication only: 44%,  Stent: 35%, CABG: 21% | Angina treatment-  Medication only: 37%,  Stent: 49%, CABG: 14% | 75% | 80.40% |
| **Forman, 2014** | mean age: 59 33% >65 years | no  control  group | 77% | no  control  group |  | no control group | 90% | no control group |
| **Kraal, 2014** | 60.6 +/- 7.5 | 56.1 +/- 8.7 | 88% | 84% | ACS with PCI:56%, ACS without PCI:16%, AP with PCI:8%, AP without PCI:8%, CABG:12% | ACS with PCI:40%, ACS without PCI:20%, AP with PCI:16%, AP without PCI:0 %, CABG:24% | 86.20% | 96.20% |
| **Piotrowicz, 2014** | 58.3 +/- 10.5 | no  control  group | 84% | no  control  group | Post an acute event: 85%, Chronic:15%, Coronary artery disease: 91%, Myocardial infarction: 72%, PCI: 62%, CABG: 24% | no control group | 99.20% | no control group |
| **Varnfield, 2014** | 54.9 +/- 9.6 | 56.2 +/- 10.1 | 91% | 83% | STEMI:49%, NSTEMI:49%, Other:2% | STEMI:56%, NSTEMI:44%, Other:0% | 86.80% | 63.40% |
| **Whittaker, 2014** |  |  |  |  |  |  | 80% | 47% |
| **Dale, 2015** | 59.0 (10.5) | 59.9 (11.8) | 79% | 84% | PCI: 70%, CABG: 23%, Medical management: 7% | PCI: 76%, CABG: 16%,  Medical management: 8% | 93.40% | 95.20% |
| **Frederix, 2015** | 58 +/- 9 | 63 +/- 10 | 81% | 85% | CABG: 23%, PCI:77% | CABG: 29%, PCI:71% | 80% | 85% |
| **Lear, 2015** | 61.7 (51.3, 65.2) | 58.4 (52.8, 64.7) | 90% | 80% | Education: Less Than High School: 21%,  High School: 26%,  Some Post-Secondary: 24%, Post-Secondary Degree/Diploma: 24%,  Post-Graduate Degree: 5%  MI: 45%, CABG: 29%, Angioplasty: 55%, Other Heart Condition: 29% | Education: Less Than High School: 23%, High School: 20%, Some Post-Secondary: 23%, Post-Secondary Degree/Diploma:25%, Post-Graduate Degree:10%  MI:48% CABG:18%, Angioplasty:60%, Other Heart Condition:10% | 100% | 100% |
| **Maddison, 2015** | 61.4 (8.9) | 59.0 (9.5) | 81% | 81% |  |  | 88.20% | 90.70% |
| **Smolis-Bak, 2015** | 60.0 +/- 8.5 | 65.1 +/- 8.2 | 96.10% | 84.60% | CHF of ischemic or another etiology, NYHA class III and implanted CRT-D | CHF of ischemic or another etiology, NYHA class III and implanted CRT-D |  |  |
| **Frederix, 2016** | 61 +/- 9 | 61 +/- 8 | 86% | 79% | Severity of disease:  NYHA I: 78%, NYHA II: 18%, NYHA III: 4% | Severity of disease:  NYHA I: 87%, NYHA II: 6%, NYHA III: 7% | 89.90% | 91.40% |
| **Skobel, 2016** | 60 (50,65) | 58 (52, 67) | 91% | 87% |  |  | 21.80% | 66.70% |
| **Thorup, 2016** | 62.8 |  | 80% |  | ACS:48%, Heart failure:11%, ACS + HF: 8% Surgery: 33% |  | 88.90% | 79.70% |
| **da Silva Vieira, 2017** | Intervention Group (IG) 1:  55 +/- 9.0,  IG2: 59 +/- 11.3 | 59 5.8 |  |  | ACS without ST elevation: IG1=55%, IG2=55%, ACS with ST elevation: IG1=45%, IG2=27%, Stable Angina Pectoris and post-angioplasty: IG1=0%, IG2=18%, Low cardiovascular risk: IG1=64%, IG2=64%, Moderate cardiovascular risk: IG1=36%, IG2=36% | ACS without ST elevation: 45%, ACS with ST elevation: 55%, Stable Angina Pectoris and post-angioplasty: 0%, Low Cardiovascular risk: 73%, Moderate cardiovascular risk: 27% | IG1:73.3%, IG2:73.3% | 68.80% |
| **Hwang, 2017** | 68 (14) | 67 (11) | 79% | 72% | NYHA I: 13%, NYHA II: 37%, NYHA III: 50%, NYHA IV: 0% | NYHA I: 7%, NYHA II: 72%, NYHA III: 21%, NYHA IV: 0% | 95.80% | 89.70% |
| **Fang, 2018** | 60.24 +/- 9.351 | 61.41 +/- 10.169 | 63.60% | 61.80% | Education: primary or less: 45.5%,  middle school: 33.3%,  high school or above: 21.2%.  Severity of disease: class I: 12.1%,  class II: 60.6%, class III: 27.3% | Education: primary or less: 50%, middle school :29.4%, high school or above:20.6% | 82.50% | 85% |
| **Harzand, 2018** | 65 +/- 5 | no  control  group | 100% | no  control  group |  | no control group | 72.20% | no control group |
| **Maddison, 2018** | 61.0 +/- 13.2 | 61.5 +/- 12.2 | 84.20% | 87.50% | Angina pectoris: 40.2%, MI: 74.4%, Angioplasty: 65.9%, CABG: 20.7% | Angina pectoris: 43.8%, MI: 75%, Angioplasty: 63.8%, CABG: 27.5% | 79.30% | 86.30% |
| **Peng, 2018** | age<60:14 (28.6%), age>60:35 (71.4%) | age <60:16 (32.7%), age>60:33 (67.3%) | 57.10% | 61.20% | Education: Junior high school or below: 73.5%, Senior high school or above: 26.5% NYHA I: 22.4%, NYHA II: 36.7%, NYHA III: 40.8% | Education:  Junior high school or below: 69.4%,  Senior high school or above: 30.6%  NYHA I: 26.5%, NYHA II: 36.7%, NYHA III: 36.7% | 85.70% | 83.70% |
| **Rawstorn, 2018** | 61.9 (36.0-85.0) |  | 84.15% |  | Angina pectoris: 40.24%,  MI: 74.39%, Angioplasty: 65.85%, CABG: 20.73% |  | 79.30% | 86.30% |

This is a Multimedia Appendix to a full manuscript published in the J Med Internet Res. For full copyright and citation information see <https://dx.doi.org/10.2196/jmir.18773>
