# Supplementary material for: Digital Health Interventions for Cardiac Rehabilitation: Systematic Literature Review
Source: J Med Internet Res. 2021 Feb 8;23(2):e18773. doi: 10.2196/18773 (PMC7899799; doi:10.2196/18773)
Supplement: Multimedia Appendix 5 [file jmir_v23i2e18773_app5.docx]

**NIH Quality Assessment for Studies Reviewed**

| **Author, Year** | **Reviewer** | **Study Type** | **1** | **2** | **3** | **4** | **5** | **6** | **7** | **8** | **9** | **10** | **11** | **12** | **13** | **14** | **Overall Quality Rating** |
| --- | --- | --- | --- | --- | --- | --- | --- | --- | --- | --- | --- | --- | --- | --- | --- | --- | --- |
| **Ades, 2000** | **#1-YK** | **QA of Controlled Intervention Studies** | **Yes** | **No** | **No** | **No** | **No** | **Yes** | **Yes** | **NA** | **Yes** | **Yes** | **Yes** | **No** | **Yes** | **Yes** | **Fair** |
|  | **#2-NO** | **QA of Controlled Intervention Studies** | **No** | **NA** | **No** | **No** | **No** | **No** | **Yes** | **Yes** | **NA** | **Yes** | **Yes** | **Yes** | **Yes** | **Yes** | **Good** |
|  |  |  |  |  |  |  |  |  |  |  |  |  |  |  |  |  |  |
| **Jenny, 2001** | **#1-YK** | **QA of Controlled Intervention Studies** | **Yes** | **Yes** | **NA** | **NA** | **NA** | **Yes** | **Yes** | **NA** | **Yes** | **Yes** | **Yes** | **No** | **Yes** | **Yes** | **Fair** |
|  | **#2-NO** | **QA of Controlled Intervention Studies** | **Yes** | **Yes** | **NA** | **No** | **No** | **Yes** | **Yes** | **NA** | **Yes** | **Yes** | **Yes** | **No** | **Yes** | **Yes** | **Fair** |
|  |  |  |  |  |  |  |  |  |  |  |  |  |  |  |  |  |  |
| **Gordon, 2002** | **#1- YK** | **QA of Controlled Intervention Studies** | **Yes** | **Yes** | **No** | **No** | **No** | **Yes** | **Yes** | **Yes** | **Yes** | **Yes** | **Yes** | **No** | **Yes** | **Yes** | **Good** |
|  | **#2- NO** | **QA of Controlled Intervention Studies** | **Yes** | **NA** | **NA** | **No** | **NA** | **Yes** | **Yes** | **NA** | **NA** | **Yes** | **Yes** | **No** | **Yes** | **Yes** | **Good** |
|  |  |  |  |  |  |  |  |  |  |  |  |  |  |  |  |  |  |
| **Southard 2003** | **#1- YK** | **QA of Controlled Intervention Studies** | **Yes** | **Yes** | **No** | **No** | **No** | **Yes** | **Yes** | **NA** | **Yes** | **Yes** | **Yes** | **No** | **Yes** | **Yes** | **Good** |
|  | **#2- NO** | **QA of Controlled Intervention Studies** | **Yes** | **Yes** | **Yes** | **No** | **No** | **Yes** | **Yes** | **Yes** | **Yes** | **Yes** | **Yes** | **No** | **Yes** | **Yes** | **Good** |
|  |  |  |  |  |  |  |  |  |  |  |  |  |  |  |  |  |  |
| **Barnason, 2009** | **#1- YK** | **QA of Controlled Intervention Studies** | **Yes** | **Yes** | **NA** | **NA** | **NA** | **Yes** | **No** | **NA** | **Yes** | **Yes** | **Yes** | **Yes** | **Yes** | **Yes** | **Good** |
|  | **#2-NO** | **QA of Controlled Intervention Studies** | **Yes** | **Yes** | **No** | **No** | **No** | **No** | **Yes** | **Yes** | **Yes** | **NA** | **Yes** | **Yes** | **Yes** | **Yes** | **Good** |
|  |  |  |  |  |  |  |  |  |  |  |  |  |  |  |  |  |  |
| **Scalvini,2009** | **#1- YK** | **QA for Observational Cohort and Cross-Sectional Studies** | **Yes** | **Yes** | **NA** | **Yes** | **No** | **NA** | **Yes** | **NA** | **Yes** | **Yes** | **Yes** | **Yes** | **Yes** | **NA** | **Fair** |
|  | **#2-NO** | **QA for Observational Cohort and Cross-Sectional Studies** | **Yes** | **Yes** | **Yes** | **Yes** | **Yes** | **Yes** | **No** | **Yes** | **Yes** | **NA** | **Yes** | **NA** | **Yes** | **NA** | **Good** |
|  |  |  |  |  |  |  |  |  |  |  |  |  |  |  |  |  |  |
| **Piotrowicz, 2010** | **#1- YK** | **QA of Controlled Intervention Studies** | **Yes** | **Yes** | **NA** | **NA** | **NA** | **Yes** | **NA** | **NA** | **Yes** | **Yes** | **Yes** | **No** | **Yes** | **Yes** | **Fair** |
|  | **#2-NO** | **QA of Controlled Intervention Studies** | **No** | **NA** | **NA** | **NA** | **NA** | **NA** | **NA** | **NA** | **No** | **NA** | **Yes** | **No** | **No** | **Yes** | **Poor** |
|  |  |  |  |  |  |  |  |  |  |  |  |  |  |  |  |  |  |
| **Reid, 2011** | **#1- YK** | **QA of Controlled Intervention Studies** | **Yes** | **Yes** | **Yes** | **NA** | **Yes** | **Yes** | **No** | **NA** | **Yes** | **Yes** | **Yes** | **No** | **Yes** | **Yes** | **Good** |
|  | **#2-NO** | **QA of Controlled Intervention Studies** | **Yes** | **Yes** | **Yes** | **Yes** | **Yes** | **Yes** | **Yes** | **No** | **No** | **Yes** | **Yes** | **Yes** | **Yes** | **Yes** | **Good** |
|  |  |  |  |  |  |  |  |  |  |  |  |  |  |  |  |  |  |
| **Clark, 2013** | **#1- YK** | **QA for Observational Cohort and Cross-Sectional Studies** | **Yes** | **Yes** | **Yes** | **Yes** | **No** | **No** | **Yes** | **Yes** | **NA** | **Yes** | **Yes** | **NA** | **NA** | **NA** | **Fair** |
|  | **#2-NO** | **QA for Observational Cohort and Cross-Sectional Studies** | **Yes** | **Yes** | **Yes** | **Yes** | **No** | **No** | **Yes** | **NA** | **Yes** | **No** | **Yes** | **No** | **Yes** | **No** | **Good** |
|  |  |  |  |  |  |  |  |  |  |  |  |  |  |  |  |  |  |
| **Brough, 2014** | **#1- YK** | **QA for Observational Cohort and Cross-Sectional Studies** | **Yes** | **Yes** | **Yes** | **Yes** | **No** | **NA** | **Yes** | **Yes** | **Yes** | **Yes** | **Yes** | **NA** | **Yes** | **NA** |  |
|  | **#2-NO** | **QA for Observational Cohort and Cross-Sectional Studies** | **Yes** | **Yes** | **Yes** | **Yes** | **No** | **Yes** | **Yes** | **Yes** | **Yes** | **Yes** | **Yes** | **No** | **Yes** | **No** | **Fair** |
|  |  |  |  |  |  |  |  |  |  |  |  |  |  |  |  |  |  |
| **Devi, 2014** | **#1-YK** | **QA of Controlled Intervention Studies** | **Yes** | **Yes** | **Yes** | **No** | **No** | **Yes** | **Yes** | **No** | **Yes** | **Yes** | **Yes** | **Yes** | **Yes** | **Yes** | **Good** |
|  | **#2-NO** | **QA of Controlled Intervention Studies** | **Yes** | **Yes** | **Yes** | **No** | **No** | **Yes** | **Yes** | **No** | **Yes** | **Yes** | **Yes** | **Yes** | **Yes** | **Yes** | **Good** |
|  |  |  |  |  |  |  |  |  |  |  |  |  |  |  |  |  |  |
| **Forman,2014** | **#1- YK** | **QA for Observational Cohort and Cross-Sectional Studies** | **Yes** | **Yes** | **Yes** | **Yes** | **No** | **NA** | **Yes** | **Yes** | **Yes** | **Yes** | **Yes** | **NA** | **Yes** | **Yes** | **Good** |
|  | **#2-NO** | **QA for Observational Cohort and Cross-Sectional Studies** | **Yes** | **Yes** | **No** | **Yes** | **No** | **No** | **Yes** | **No** | **Yes** | **No** | **Yes** | **No** | **No** | **No** | **Fair** |
|  |  |  |  |  |  |  |  |  |  |  |  |  |  |  |  |  |  |
| **Kraal, 2014** | **#1-YK** | **QA of Controlled Intervention Studies** | **Yes** | **Yes** | **Yes** | **No** | **NA** | **Yes** | **Yes** | **NA** | **Yes** | **Yes** | **Yes** | **NA** | **Yes** | **Yes** | **Good** |
|  | **#2-NO** | **QA of Controlled Intervention Studies** | **Yes** | **Yes** | **Yes** | **Yes** | **NA** | **Yes** | **Yes** | **Yes** | **Yes** | **Yes** | **Yes** | **No** | **Yes** | **Yes** | **Fair** |
|  |  |  |  |  |  |  |  |  |  |  |  |  |  |  |  |  |  |
| **Piotrowicz, 2014** | **#1- YK** | **QA of Controlled Intervention Studies** | **No** | **NA** | **NA** | **No** | **NA** | **NA** | **Yes** | **NA** | **Yes** | **Yes** | **Yes** | **No** | **Yes** | **NA** | **Fair** |
|  | **#2-NO** | **QA of Controlled Intervention Studies** | **Yes** | **NA** | **NA** | **NA** | **NA** | **Yes** | **Yes** | **Yes** | **Yes** | **Yes** | **Yes** | **No** | **Yes** | **Yes** | **Good** |
|  |  |  |  |  |  |  |  |  |  |  |  |  |  |  |  |  |  |
| **Varnfield, 2014** | **#1-YK** | **QA of Controlled Intervention Studies** | **Yes** | **Yes** | **NA** | **No** | **NA** | **Yes** | **Yes** | **NA** | **Yes** | **Yes** | **Yes** | **NA** | **Yes** | **Yes** | **Good** |
|  | **#2-NO** | **QA of Controlled Intervention Studies** | **Yes** | **Yes** | **Yes** | **No** | **Yes** | **No** | **No** | **No** | **No** | **Yes** | **Yes** | **Yes** | **No** | **Yes** | **Fair** |
|  |  |  |  |  |  |  |  |  |  |  |  |  |  |  |  |  |  |
| **Whittaker, 2014** | **#1- YK** | **QA of Controlled Intervention Studies** | **Yes** | **NA** | **NA** | **NA** | **NA** | **Yes** | **Yes** | **NA** | **Yes** | **Yes** | **Yes** | **No** | **No** | **NA** | **Fair** |
|  | **#2-NO** | **QA of Controlled Intervention Studies** | **No** | **NA** | **NA** | **NA** | **NA** | **Yes** | **Yes** | **NA** | **Yes** | **Yes** | **Yes** | **No** | **No** | **NA** | **Fair** |
|  |  |  |  |  |  |  |  |  |  |  |  |  |  |  |  |  |  |
| **Dale, 2015** | **#1-YK** | **QA for Observational Cohort and Cross-Sectional Studies** | **Yes** | **Yes** | **Yes** | **No** | **NA** | **Yes** | **NA** | **NA** | **Yes** | **Yes** | **Yes** | **Yes** | **Yes** | **Yes** | **Good** |
|  | **#2- NO** | **QA for Observational Cohort and Cross-Sectional Studies** | **Yes** | **Yes** | **Yes** | **No** | **Yes** | **Yes** | **No** | **Yes** | **Yes** | **Yes** | **Yes** | **Yes** | **Yes** | **Yes** | **Good** |
|  |  |  |  |  |  |  |  |  |  |  |  |  |  |  |  |  |  |
| **Frederix, 2015** | **#1- YK** | **QA of Controlled Intervention Studies** | **Yes** | **Yes** | **No** | **No** | **NA** | **Yes** | **Yes** | **NA** | **Yes** | **Yes** | **Yes** | **Yes** | **Yes** | **Yes** | **Good** |
|  | **#2- NO** | **QA of Controlled Intervention Studies** | **Yes** | **No** | **Yes** | **Yes** | **NA** | **Yes** | **Yes** | **Yes** | **Yes** | **Yes** | **Yes** | **Yes** | **NA** | **NA** | **Poor** |
|  |  |  |  |  |  |  |  |  |  |  |  |  |  |  |  |  |  |
| **Lear, 2015** | **#1-YK** | **QA of Controlled Intervention Studies** | **Yes** | **Yes** | **NA** | **No** | **NA** | **Yes** | **NA** | **NA** | **NA** | **Yes** | **Yes** | **No** | **Yes** | **Yes** | **Good** |
|  | **#2-NO** | **QA of Controlled Intervention Studies** | **Yes** | **Yes** | **No** | **No** | **NA** | **Yes** | **Yes** | **Yes** | **Yes** | **Yes** | **Yes** | **Yes** | **Yes** | **Yes** | **Good** |
|  |  |  |  |  |  |  |  |  |  |  |  |  |  |  |  |  |  |
| **Maddison, 2015** | **#1- YK** | **QA of Controlled Intervention Studies** | **Yes** | **Yes** | **NA** | **NA** | **NA** | **Yes** | **NA** | **NA** | **Yes** | **Yes** | **Yes** | **NA** | **Yes** | **Yes** | **Good** |
|  | **#2-NO** | **QA of Controlled Intervention Studies** | **Yes** | **Yes** | **Yes** | **Yes** | **Yes** | **Yes** | **Yes** | **Yes** | **NA** | **Yes** | **Yes** | **Yes** | **Yes** | **Yes** | **Good** |
|  |  |  |  |  |  |  |  |  |  |  |  |  |  |  |  |  |  |
| **Smolis-Bak, 2015** | **#1-YK** | **QA of Controlled Intervention Studies** | **No** | **Yes** | **No** | **No** | **No** | **Yes** | **NA** | **NA** | **Yes** | **Yes** | **Yes** | **NA** | **Yes** | **Yes** | **Good** |
|  | **#2-NO** | **QA of Controlled Intervention Studies** | **Yes** | **Yes** | **No** | **No** | **No** | **Yes** | **NA** | **NA** | **Yes** | **Yes** | **Yes** | **NA** | **Yes** | **Yes** | **Good** |
|  |  |  |  |  |  |  |  |  |  |  |  |  |  |  |  |  |  |
| **Frederix, 2016** | **#1-YK** | **QA of Controlled Intervention Studies** | **Yes** | **Yes** | **No** | **No** | **NA** | **Yes** | **Yes** | **NA** | **Yes** | **Yes** | **Yes** | **Yes** | **Yes** | **Yes** | **Good** |
|  | **#2- NO** | **QA of Controlled Intervention Studies** | **Yes** | **Yes** | **Yes** | **No** | **NA** | **Yes** | **Yes** | **Yes** | **Yes** | **Yes** | **Yes** | **Yes** | **Yes** | **Yes** | **Good** |
|  |  |  |  |  |  |  |  |  |  |  |  |  |  |  |  |  |  |
| **Skobel, 2016** | **#1- YK** | **QA of Controlled Intervention Studies** | **Yes** | **Yes** | **NA** | **NA** | **NA** | **Yes** | **No** | **NA** | **No** | **Yes** | **Yes** | **No** | **Yes** | **Yes** | **Fair** |
|  | **#2 -NO** | **QA of Controlled Intervention Studies** | **Yes** | **Yes** | **No** | **No** | **Yes** | **Yes** | **No** | **No** | **Yes** | **Yes** | **Yes** | **No** | **Yes** | **Yes** | **Good** |
|  |  |  |  |  |  |  |  |  |  |  |  |  |  |  |  |  |  |
| **Thorup, 2016** | **#1-YK** | **QA of Controlled Intervention Studies** | **Yes** | **Yes** | **No** | **No** | **NA** | **Yes** | **NA** | **NA** | **Yes** | **NA** | **Yes** | **NA** | **NA** | **Yes** | **Fair** |
|  | **#2- NO** | **QA of Controlled Intervention Studies** | **Yes** | **Yes** | **NA** | **No** | **No** | **No** | **Yes** | **Yes** | **Yes** | **Yes** | **Yes** | **No** | **Yes** | **NA** | **Good** |
|  |  |  |  |  |  |  |  |  |  |  |  |  |  |  |  |  |  |
| **da Silva Vieira, 2017** | **#1-YK** | **QA of Controlled Intervention Studies** | **Yes** | **Yes** | **NA** | **NA** | **NA** | **Yes** | **No** | **NA** | **Yes** | **Yes** | **Yes** | **Yes** | **Yes** | **Yes** | **Good** |
|  | **#2- NO** | **QA of Controlled Intervention Studies** | **Yes** | **Yes** | **No** | **No** | **No** | **Yes** | **Yes** | **Yes** | **Yes** | **Yes** | **Yes** | **Yes** | **Yes** | **Yes** | **Good** |
|  |  |  |  |  |  |  |  |  |  |  |  |  |  |  |  |  |  |
| **Hwang, 2017** | **#1-YK** | **QA of Controlled Intervention Studies** | **Yes** | **Yes** | **Yes** | **No** | **NA** | **Yes** | **Yes** | **NA** | **Yes** | **Yes** | **Yes** | **No** | **Yes** | **Yes** | **Good** |
|  | **#2 -NO** | **QA of Controlled Intervention Studies** | **Yes** | **NA** | **Yes** | **NA** | **Yes** | **Yes** | **Yes** | **Yes** | **Yes** | **Yes** | **Yes** | **Yes** | **Yes** | **Yes** | **Good** |
|  |  |  |  |  |  |  |  |  |  |  |  |  |  |  |  |  |  |
| **Fang, 2018** | **#1-YK** | **QA of Controlled Intervention Studies** | **Yes** | **Yes** | **No** | **No** | **No** | **Yes** | **No** | **No** | **Yes** | **Yes** | **Yes** | **NA** | **Yes** | **Yes** | **Good** |
|  | **#2-NO** | **QA of Controlled Intervention Studies** | **Yes** | **NA** | **NA** | **NA** | **NA** | **Yes** | **Yes** | **No** | **Yes** | **Yes** | **Yes** | **NA** | **Yes** | **Yes** |  |
|  |  |  |  |  |  |  |  |  |  |  |  |  |  |  |  |  |  |
| **Harzand, 2018** | **#1- YK** | **QA for Observational Cohort and Cross-Sectional Studies** | **Yes** | **Yes** | **Yes** | **Yes** | **No** | **NA** | **Yes** | **Yes** | **Yes** | **Yes** | **Yes** | **NA** | **Yes** | **NA** | **Good** |
|  | **#2- NO** | **QA for Observational Cohort and Cross-Sectional Studies** | **No** | **NA** | **No** | **No** | **No** | **Yes** | **Yes** | **NA** | **Yes** | **Yes** | **Yes** | **No** | **NA** | **NA** | **Good** |
|  |  |  |  |  |  |  |  |  |  |  |  |  |  |  |  |  |  |
| **Maddison, 2018** | **#1- YK** | **QA of Controlled Intervention Studies** | **Yes** | **Yes** | **Yes** | **No** | **Yes** | **Yes** | **Yes** | **No** | **Yes** | **Yes** | **Yes** | **NA** | **Yes** | **Yes** | **Good** |
|  | **#2-NO** | **QA of Controlled Intervention Studies** | **Yes** | **NA** | **Yes** | **No** | **Yes** | **Yes** | **Yes** | **Yes** | **Yes** | **Yes** | **Yes** | **Yes** | **Yes** | **Yes** | **Good** |
|  |  |  |  |  |  |  |  |  |  |  |  |  |  |  |  |  |  |
| **Peng, 2018** | **#1-YK** | **QA of Controlled Intervention Studies** | **Yes** | **Yes** | **Yes** | **NA** | **Yes** | **Yes** | **Yes** | **NA** | **Yes** | **Yes** | **Yes** | **Yes** | **Yes** | **Yes** | **Good** |
|  | **#2-NO** | **QA of Controlled Intervention Studies** | **Yes** | **Yes** | **Yes** | **NA** | **Yes** | **Yes** | **Yes** | **Yes** | **Yes** | **Yes** | **Yes** | **Yes** | **Yes** | **Yes** | **Good** |
|  |  |  |  |  |  |  |  |  |  |  |  |  |  |  |  |  |  |
| **Rawstom, 2018** | **#1- YK** | **QA of Controlled Intervention Studies** | **Yes** | **Yes** | **NA** | **NA** | **NA** | **Yes** | **No** | **NA** | **Yes** | **Yes** | **Yes** | **No** | **Yes** | **Yes** | **Fair** |
|  | **#2-NO** | **QA of Controlled Intervention Studies** | **Yes** | **NA** | **NA** | **NA** | **NA** | **Yes** | **Yes** | **Yes** | **No** | **Yes** | **Yes** | **No** | **No** | **No** | **Poor** |

**Abbreviation:** QA: quality assessment

Numbers 1-14 indicate question numbers as detailed in the Supplemental Table 1 and Supplemental Table 2.

This is a Multimedia Appendix to a full manuscript published in the J Med Internet Res. For full copyright and citation information see <https://dx.doi.org/10.2196/jmir.18773>
