# Supplementary material for: Digital Health Interventions for Cardiac Rehabilitation: Systematic Literature Review
Source: J Med Internet Res. 2021 Feb 8;23(2):e18773. doi: 10.2196/18773 (PMC7899799; doi:10.2196/18773)
Supplement: Multimedia Appendix 6 [file jmir_v23i2e18773_app6.docx]

**Aspects of In-Person Sessions**

| **Author, Year** | **In-Person Sessions** |
| --- | --- |
| **Ades, 2000** | BA: baseline graded symptom-limited exercise test with ECG monitoring and expired gas analysis |
| **Jenny, 2001** | BA: clinical manifestations (e.g., severity of breathlessness on exertion, angina, severity of arrhythmia, heart rate, and blood pressure) and demographic variables |
| **Gordon, 2002** | Physician-supervised, nurse-case-managed program: baseline testing, education on CVD, CAD risk factors and lifestyle modifications, update of exercise prescription, medication changes, physician referrals, implementation of individualized nutrition, weight management, stress management and smoking cessation programs, measurement of fasting serum lipids and lipoproteins  Community-based program: baseline testing, education on CVD, CAD risk factors and lifestyle modifications, update of exercise prescription, implementation of individualized nutrition, weight management, stress management and smoking cessation programs, medication changes |
| **Southard, 2003** | BA: height, weight, BP, blood tests |
| **Barnason, 2009** | BA: demographic and clinical characteristics, MOS SF-36 Usual care (in addition to intervention) |
| **Scalvini, 2009** | BA: routine blood tests, ECG, cardiac echo color doppler scans, 6MWT Follow-up hospital visits (routine blood tests, cardiology examinations, cardiac echo color doppler scans) Home visits by nurse/physiotherapist (at least once per week) |
| **Piotrowicz, 2010** | BA: clinical examination with symptom evaluation (NYHA class), two-dimensional echocardiography, 6MWT, cardiopulmonary exercise treadmill test, and health-related quality of life  3-6 monitored educational ET sessions (during hospitalization), education (how to measure BP, HR, weight, how to perform ET, how to operate the telemonitoring equipment, self-evaluation of worrying signs |
| **Reid, 2011** | BA: questionnaire about education, leisure time physical activity, health-related QoL presentation of a  During hospitalization: instructions on how to access CardioFit website and personally tailored physical activity plan by an exercise specialist |
| **Clark, 2013** | BA, designing of a tailored program for each patient, website use training |
| **Brough, 2014** | BA: assessment of baseline characteristics, exercise performance, health status and health behaviors |
| **Devi, 2014** | BA: BP, weight, body fat percentage, questionnaire Introductory session: how to use the program |
| **Forman, 2014** | Participation in traditional CR program |
| **Kraal, 2014** | BA: maximal exercise test with respiratory gas analysis, health-related QoL questionnaire, BMI, clinical characteristics Lifestyle change therapy, relaxation and stress management education Three supervised training sessions for familiarization with training program and instructions on how to use technologies |
| **Piotrowicz, 2014** | BA: clinical examination, 6-MWT, exercise treadmill or cycloergometer test) Initial stage: optimization of treatment, education, individual planning of ET Initial monitored educational training sessions |
| **Varnfield, 2014** | BA: demographics, previous clinical history and procedural data, risk factor assessment to set individualized goals for lifestyle behavior modification, clinical assessment Training on technology use |
| **Whittaker, 2014** | BA: demographic and previous clinical, procedural and risk factor characteristics, baseline measurements |
| **Dale, 2015** | BA Access to usual care (supervised exercise program, education on CVD, lifestyle change and psychosocial support) |
| **Frederix, 2015** | BA Phase II CR: education about core components of CR, psychosocial management, physical activity counseling  Exercise in hospital's rehabilitation center using outpatient service for first 6 weeks in addition to intervention |
| **Lear, 2015** | BA (demographics, medical history, blood tests, BP, weight, waist circumference, smoking status, diet, questionnaire about physical activity), training session on the use of vCRP, HR monitor and BP monitor |
| **Maddison, 2015** | BA: demographics, respiratory gas analysis during a standardized treadmill exercise testing protocol, BP, BMI, waist circumference questionnaires about physical activity, self-efficacy, health-related QoL) Access to usual care (education on CVD risk factors, psychological support, supervised exercise) |
| **Smolis-Bak, 2015** | BA: medical history, weight, BMI, spiroergometric parameters Exercise training (at the rehabilitation center for 3 weeks) Cardiopulmonary exercise testing on treadmill Standard echocardiography (2D Doppler) 6MWT Instructions in self-assessment of their health status, operation of the telemonitoring equipment and correct performance of exercises, questionnaires about QoL and depressive symptoms |
| **Frederix, 2016** | BA: demographics, clinical characteristics, cardiopulmonary exercise testing, BMI Usual care in addition to intervention for pluridisciplinary rehabilitation sessions, exercise training, familiarization with motion sensor and web service |
| **Skobel, 2016** | BA: history, physical examination, ECG, laboratory analysis, 2D echocardiography and exercise testing with additional lactate samples, QoL questionnaire |
| **Thorup, 2016** | BA (demographics, clinical characteristics, BMI, instructions on how to use the technologies), ensuring the right use of the technologies (1 home visit) |
| **da Silva Vieira, 2017** | BA: clinical characteristics, height, BMI, body fat percentage, waist-to-hip ratio, waist-to-height ratio, blood tests, physical activity Instruction on technology use and set up |
| **Hwang, 2017** | BA: demographics, clinical characteristics, BMI, BP, HR Equipment familiarization session |
| **Fang, 2018** | BA: demographics, medical history, BP, HR, blood tests, 6MWT, CDS score, FTND score, SF36 score Home visits to enhance training |
| **Harzand, 2018** | BA: demographics, clinical characteristics, symptom-limited ETT or 6MWT, HR, BP |
| **Maddison, 2018** | BA: demographics, clinical characteristics, BP, anthropometry, blood tests, health-related QoL, treadmill cardiopulmonary exercise tests, assessment of physical activity using an accelerometer, questionnaires about exercise-related task and barrier self-efficacy Access to usual care CR |
| **Peng, 2018** | BA: demographics, questionnaire about QoL, 6MWD, HR, 2-dimensional echocardiography, NYHA classification, levels of anxiety and depression Usual care with regular follow-up visits at the clinic |
| **Rawstorn, 2018** | BA: demographics, clinical characteristics  Standard treatment (in addition to intervention) |

Abbreviations: BA: Baseline Assessment; ECG: electrocardiogram; CVD: cardiovascular disease; CAD: coronary artery disease; MOS SF-36: Medical Outcomes Study Short Form-36; 6MWT: 6-minute walking test; NYHA: New York Heart Association; ET: exercise training; BMI: body mass index; BP: blood pressure; HR: heart rate; vCRP: virtual cardiac rehabilitation program; FTND: Fagerstrom Test for Nicotine Dependence; CDS: Cardiac Depression Scale; SF36: SF-36 Health Survey; ETT: exercise treadmill test

This is a Multimedia Appendix to a full manuscript published in the J Med Internet Res. For full copyright and citation information see <https://dx.doi.org/10.2196/jmir.18773>
