# Supplementary material for: Digital Health Interventions for Cardiac Rehabilitation: Systematic Literature Review
Source: J Med Internet Res. 2021 Feb 8;23(2):e18773. doi: 10.2196/18773 (PMC7899799; doi:10.2196/18773)
Supplement: Multimedia Appendix 7 [file jmir_v23i2e18773_app7.docx]

**Technology Utilized in Studies**

| **Author, Year** | **Intervention** | **Technology Utilized** |
| --- | --- | --- |
| **Ades, 2000** | Home-based, transtelephonically monitored cardiac rehabilitation | Telerehabilitation system, bipolar ECG, ECG transmitter unit, headset, voice transmitter, telephone modem |
| **Jenny, 2001** | Interactive multimedia computer-based patient education program in cardiac rehabilitation | Computer software delivered on PC workstation or laptop: interactive tutorial for education about exercise |
| **Gordon, 2002** | Physician supervised, nurse-case-managed cardiovascular risk reduction program and community-based cardiovascular risk reduction program guided by a computerized participant management system | Physician supervised, nurse-case-managed: telephone, audiotapes Community-based: computerized participant management and tracking system, telephone, audiotapes |
| **Southard, 2003** | Internet-based cardiac rehabilitation | Internet-based program (secure website), online discussion group (between the participants), interaction between participants and case manager and dietician via secure communication lines within the program (a format similar to e-mail), telephone |
| **Barnason, 2009** | Symptom management telehealth intervention | Accelerometer, Health Buddy device |
| **Scalvini, 2009** | Home-based exercise rehabilitation with telemedicine | video-recorders (cassette or DVD) for physiotherapy, 1-lead telephonic ECG devices |
| **Piotrowicz, 2010** | Home-based telemonitored cardiac rehabilitation | ECG monitoring w/ event Holter and transmission via mobile phone |
| **Reid, 2011** | Internet-based activity prescription with online coaching | Secure website for activity planning and tracking, pedometer |
| **Clark, 2013** | Internet-based electronic outpatient cardiac rehabilitation | Web-based platform, email, telephone, digital BP machine, glucometer, pedometer |
| **Brough, 2014** | Interactive web-based program | Website, telephone, email, private messaging, exercise e-diary |
| **Devi, 2014** | Web-based program for physical activity | Accelerometer, web program, online exercise diary, email, private messaging |
| **Forman, 2014** | Mobile smartphone application for cardiac rehabilitation | Mobile device, Health Coach application, web-based dashboard |
| **Kraal, 2014** | Home-based training with telemonitoring | HR monitor, web application, telephone |
| **Piotrowicz, 2014** | Home-based cardiac telerehabilitation | Remote-controlled equipment for tele-ECG monitoring and supervised exercise training, event Holter and BP monitor transmitted via mobile phone |
| **Varnfield, 2014** | Smartphone-based home care model | Smartphone with preinstalled health diary and activity monitoring app, BP monitor, weight scale, web portal |
| **Whittaker, 2014** | Technology-enabled, home-based cardiac rehabilitation | Mobile phone, wellness web portal, text messaging, mobile phone step counter, Wellness Diary (mobile tool for wellness data collection and storage), video / tele-conference sessions |
| **Dale, 2015** | mHealth-delivered comprehensive cardiac rehabilitation | Text messages, website, pedometer |
| **Frederix, 2015** | Exercise training program with telemonitoring support | Accelerometer, email/SMS, online patient account |
| **Lear, 2015** | Virtual cardiac rehabilitation program | HR monitor, BP monitor, website, interactive chat forum |
| **Maddison, 2015** | Text messages and videos targeted at increasing exercise | Mobile phone, website, text messaging |
| **Smolis-Bak, 2015** | Home exercise training program w/ telemonitoring | ECG recording sent via smartphone |
| **Frederix, 2016** | Internet-based, comprehensive telerehabilitation program | Accelerometer, email/SMS |
| **Skobel, 2016** | Smartphone-guided cardiac rehabilitation training system | Remote monitoring system (sensor monitoring RR, ECG transmitting information to the medical team wirelessly via the internet on training intensity, arrhythmias, adherence to training prescriptions), smartphone |
| **Thorup, 2016** | Telerehabilitation program | Fitbit Zip to monitor daily steps, scale, sphygmomanometer, tablet with personal health record (PHR) access for health information/communication |
| **da Silva Vieira, 2017** | Virtual reality home-based cardiac rehabilitation | Kinect, HR monitor |
| **Hwang, 2017** | Group-based video telerehabilitation | Synchronous videoconferencing platform, telerehabilitation equipment (laptop computer, mobile broadband device connected to 3G wireless broadband internet, automatic sphygmomanometer, finger pulse oximeter) |
| **Fang, 2018** | Home-based cardiac telerehabilitation | Remote monitoring system (for exercise training monitoring): belt strap w/ sensor, smartphone application, computer servers, web portal |
| **Harzand, 2018** | Smartphone-enabled cardiac rehabilitation | Smartphone app, hospital-facing online dashboard for remote monitoring |
| **Maddison, 2018** | Remotely monitored exercise-based telerehabilitation | Smartphone, chest-worn wearable sensor (HR, RR, single lead ECG, accelerometry), smartphone and web apps |
| **Peng, 2018** | Home-based telehealth exercise program | HR monitor, smartphone, instant messaging, webcam, telephone |
| **Rawstorn, 2018** | Exercise-based cardiac telerehabilitation | Telerehabilitation platform (wearable sensor, mobile and web application, middleware) |

This is a Multimedia Appendix to a full manuscript published in the J Med Internet Res. For full copyright and citation information see <https://dx.doi.org/10.2196/jmir.18773>
